# Effect on the characterization of metolachlor polyurea microcapsules with urea instead of polyamines

**DOI:** 10.1080/15685551.2019.1653031

**Published:** 2019-08-17

**Authors:** Deming Li, Yan Wang, Jun Wang, Huanhuan Liu

**Affiliations:** College of Resources and Environment, Jilin Agricultural University, Changchun, P.R. China

**Keywords:** Polyurea, microcapsules, metolachlor, interfacial polymerization, urea, environmental protection

## Abstract

In this paper, a novel metolachlor microcapsules suspension (CS) was prepared by interfacial polymerization. Metolachlor polyurea microcapsules suspension was successfully prepared by urea instead of polyamines which could reduce the use of organic solvents and production costs. The synthesized microcapsules were characterized by Fourier Transform Infrared Spectrometer, Scanning Electron Microscope, Ultraviolet Spectrometry, Thermogravimetric analyses and particle size analyzer. In conclusion, the diameter of the urea microcapsules were the smallest (11.52 μm) and excellent encapsulation efficiency (81.45%). In addition, Urea microcapsules had compact microstructures and global shapes, which had a good thermal stability and metolachlor could be preserved better in the polyurea microcapsules. These results indicated that the prepared microcapsules by urea had better thermal storage properties and physicochemical property. The microcapsule suspension of metolachlor hasn’t been researched yet. Therefore, it is significant to prepare microcapsule suspension. More importantly, there is no use of organic solvents in the preparation of microcapsules suspension, which avoided the pollution of solvents to the ecological environment. It is hoped that this polyurea material will be applied in the field of pesticide synthesis and polymers.

## 1. Introduction

Metolachlor[(RS)-2-Chloro-N-(2-ethyl-6-methyl-phenyl)-N-(1-methoxypropan-2-yl)acetamide], has been used as a chloroacetanilide herbicide to control annual grass weeds and broadleaf weeds in corn, cotton, peanuts soybeans and beans [1]. It has the characteristics of strong inhibition and good safety. At present, the extensive use of pesticides has enabled significant increase of food production. Due to a large amount of pesticides application and their degradation, volatilization and leaching into environment, which can cause environmental pollution and ecological issues seriously. In particular, the main formulation of metolachlor is emulsifiable concentrate, which contains a large amount of organic solvents such as toluene and xylene. It will cause high pollution to the environment. Those organic solvents had damaged the stability and the biodiversity of ecosystem. Pesticide capsule suspensions (CS) are a new water-based sustained release formulation. It had long persistent control and 2 to 3 years of shelf life [2,3]. Pesticide microcapsule formulations can be used to decrease toxicity to mammals and extend activity. It also has been used to prevent the evaporation of volatile compounds, control the rate of release, reduce phytotoxicity, protect pesticide from rapid environmental degradation, reduce leaching and pesticide levels in the environment [3].

Microcapsules are small particles with sizes between 1 and 100 µm that contain an active agent surrounded by a natural or synthetic polymeric membrane. Microencapsulation is a method used to obtain products with controlled release properties. It is a process that allows to isolate an active product from the external medium by forming microspheres or microcapsules [4]. At present, many methods have been developed for microencapsulation including interfacial polymerization [5,6], in situ polymerization [7,8], complex coagulation and suspension polymerization [9,10]. The interfacial polycondensation has been widely concerned since it has several unique advantages, such as high reaction speed and mild reaction course [11]. The chemical properties of polyurea microcapsules containing pesticide metolachlor have not been studied. In this study, polyurea shell material was synthesized by interfacial polycondensation using urea and toluene-2,4-diisocyanate as monomers.

The method of synthesization polymers by interfacial polycondensation reaction was invented by Du Pont in the late 1950s, and the shell materials included polyamides, polyurethanes, polyureas [12], polyanilines and polyimides. Interfacial polycondensation is a widely applicable encapsulation method. It offers rapid polymer production in an almost ready-to-use form at ambient temperature and pressure [13]. In this microencapsulation process, (O/W) or (W/O) emulsion must be prepared first, where the oil soluble monomer or the water soluble monomer must be dissolved in the core liquid phase beforehand and then, the water soluble monomer (or the oil soluble monomer) is added into the continuous phase after preparation of emulsion [14–18].

The purpose of this paper is to investigate whether polyurea microcapsules can be prepared with urea. To the best of our knowledge. The method of metolachlor polyurea microcapsules by interfacial polycondensation reaction will use a large amount of polyamines and organic solvents. Replacing polyamines with urea can reduce production costs and reduce environmental pollution of organic solvents. And we also hope the work could provide some basic reference data for industrial production of metolachlor microcapsule suspension.

## 2. Experimental part

### 2.1. Materials

**Figure 1.**
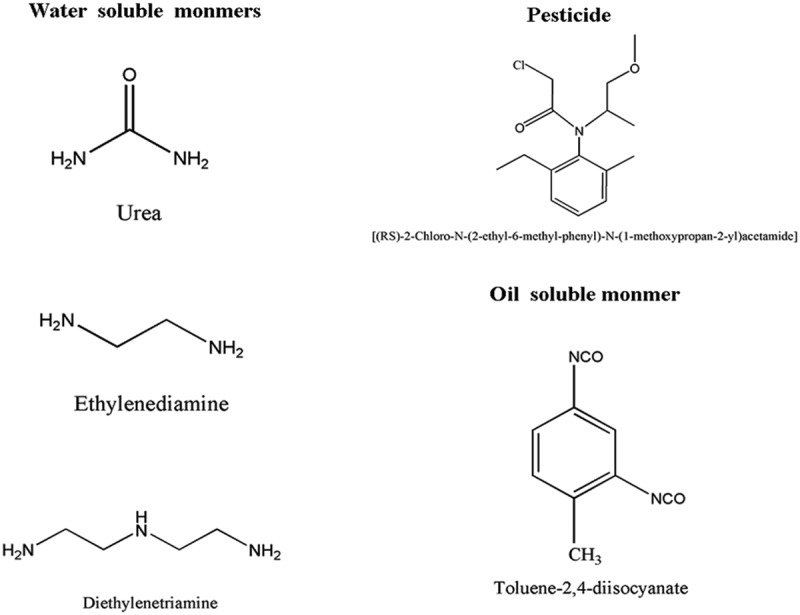
Molecular structures of core and shell materials used in this study.

Metolachlor (95% purity) used as core materials of the microcapsule was obtained from Agricultural Science and Technology of Binzhou, Shandong, China, Co., Ltd. Toluene-2,4-diisocyanate (TDI,C.P.) and Diethylenetriamine (DETA,AR) used to form shell materials were supplied by Shanghai Saen Chemical Technology Co., Ltd. Urea (AR) was from Xilong Chemical Co., Ltd. Sodium hydroxide (AR) and styrene-maleic anhydride copolymer (SMA) was purchased from Shanghai Zzbio Co., Ltd. Ethylenediamine (EDA,AR) was purchased from Beijing Chemical Works. Figure 1 shows chemical structures of Metolachlor, TDI, EDA, DETA and Urea.

### 2.2. Instruments

Homomixer (JSF-400, Shanghai Pushen Chem, China). Scanning Electron Microscope (SEM) imaging was carried out by a SSX-550 Scanning Electron Microscope (Shimadzu, Japan). Sputter Coater ETD-2000 (Elaborate Technology Development Ltd., Beijing, China) was utilized to coat a golden layer on samples. Particle size and structure were characterized by a laser particle size analyzer (BT-9300H, Dandong, China) and Fourier Transform Infrared Spectrometer (FT-IR Affinity-1, Shimadzu, Japan). Ultraviolet Spectrometry (UV-2450, Shimadzu, Japan) was used to detect the concentration of non-encapsulated metolachlor.

### 2.3. Preparation of microcapsules

**Figure 2.**
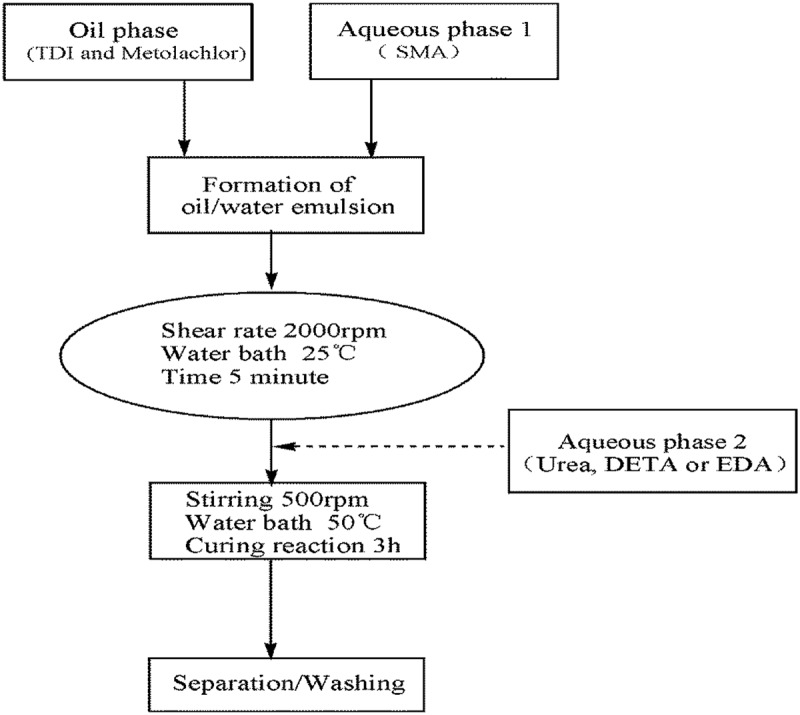
The preparation process of the polyurea microcapsules containing metolachlor.

Firstly, 1.8 g styrene-maleic anhydride copolymer (SMA) and 0.50 g Sodium hydroxide and 16 mL distilled water were added into a 100 mL flask, increasing dissolving by heating. The continuous phase was an aqueous solution of styrene-maleic anhydride copolymer. Figure 2 illustrates the preparation process of the polyurea microcapsules containing metolachlor. Table 1 shows the formulation characteristic of microcapsules containing metolachlor with different aqueous monomers.

**Table 1.** Formulation characteristic of microcapsules containing metolachlor with different aqueous monomers.

	Aqueous monomers	Metolachlor (mL)	Distilled water (mL)	SMA (g)	TDI (mL)	Monomers quality (g)	Polyurea yield(%)
a	Urea	3.16	16	1.8	2.89	1.99	85.76
b	DETA	3.16	16	1.8	2.89	3.42	80.58
c	EDA	3.16	16	1.8	2.89	1.99	81.45

**Figure 3.**
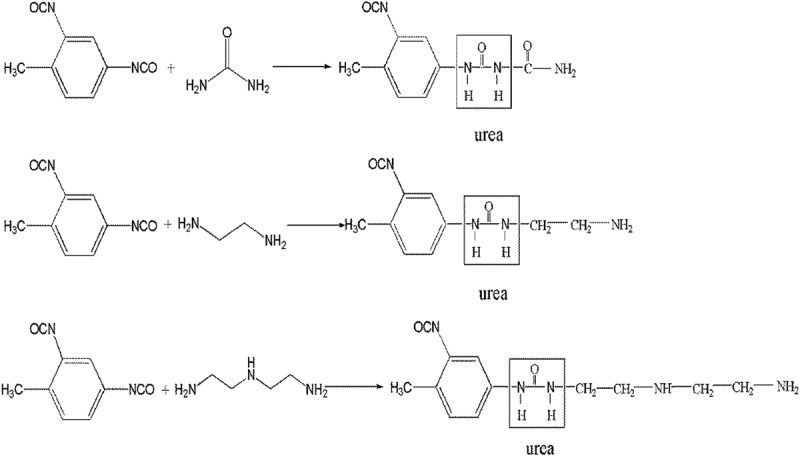
Wall-forming reaction of the polyfunctional isocyanate microcapsule system.

Then a given weight of TDI was added to the oil phase. The dispersed phase was poured into continuous water phase with 6 wt % of styrene-maleic anhydride copolymer as emulsifying agent to form oil/water emulsion. Emulsification was carried for 5 min by using homomixer (JSF-400, Shanghai Pushen Chem, China) with emulsification rate of 2000 rpm. After emulsification, the oil/water emulsion was taken into a 50 mL three-neck round-bottomed flask. The second monomer, urea or other polyamines as shown in Table 1, was added in the continuous phase after the formation of the liquid–liquid dispersion. The emulsion was stirred with certain agitation rate by using two bladed stirring paddles. The wall-forming reaction (Figure 3) is initiated by heating the oil-in-water emulsion to 50°C, at which point the isocyanate monomers are hydrolysed at the interface (slow step) to form amines, in turn, react with unhydrolysed monomers at the interface to form the polyurea microcapsule wall. The total reaction time was 3 h. The resulting microcapsules were collected by centrifugation and washed-first with 30% ethanol aqueous solution and thereafter twice with distilled water to remove the remaining reactants.

## 3. Characterization of microcapsules

### 3.1. FT-IR analysis of microcapsules

The microcapsules were dried completely, and ground with potassium bromide (KBr) for tablets. Metolachlor was daubed on potassium bromide liquid poll directly for IR analysis. Then, spectra were recorded between 400 and 4000 cm^−1^ at a resolution of 4 cm^−1^. The spectrums were modified by atmospheric correction and baseline before superposition by the installed software. The blank spectrum was prepared by using KBr.

### 3.2. Particle size measurement

The particle size and size distribution of microcapsules were measured using a laser particle size analyzer BT-9300H. All the experiments were performed under the ambient temperature. The prepared microcapsule slurries were diluted properly in water. The microcapsule particle size and size distribution were measured by the laser particle size analyzer BT-9300H. The indexes D_50_ and span are employed to describe the particle size and size distribution of microcapsules. The span was calculated according to the following Equation (1).

(1) $$span=\frac{D_{90}-D_{10}}{D_{50}}$$

where D_90_, D_10_, and D_50_ represent the diameter under the cumulative volume fractions of the microcapsules are 90%, 10%, and 50%, respectively.

### 3.3. Morphological characterization by scanning electron microscopy

The prepared polyurea microcapsules containing metolachlor samples were mounted on silicon wafers without any other treatments, and then a golden layer was sputtered on the silicon wafer using a sputter coater ETD-2000. Finally, the microcapsule samples were scanned using electron scanning electron microscopy (SSX-550, shimadzu, Japan).

### 3.4. Measurement of encapsulation efficiency

Establishment of the standard line: Scanning the ultraviolet absorption spectra of metolachlor by ultraviolet spectrophotometer. Determine the maximum absorption wavelength (λ) of metolachlor is 266 nm. Weigh 0.1052 g (accurate to 0.0001 mg) of 95% metolachlor with an electronic analytical balance. Dissolve in methanol (AR), then transfer to a 100 mL volumetric flask, constant volume, obtain 1000 ppm of mother liquor after shaking, and then pipette different volumes of mother liquor from 1000 ppm of mother liquor, respectively, to 2 ppm, 4 ppm, 6 ppm, 8 ppm, 10 ppm, 20 ppm. The standard solutions of six different concentrations of standard solutions were measured at 266 nm and a standard curve was created based on these data.

Determination of encapsulation efficiency: Firstly, 5 g anhydrous sodium sulfate and 2 g Chromatographed silicagel, which were successively filled in a column (20 × 200 mm), then 0.20 g metolachlor microcapsules suspension was added in the column with 100 mL methanol to rinse non-encapsulated metolachlor. UV-2450 Shimadzu was utilized to detect the concentration of leachate. EE was calculated via the following Equation (2).

(2) $$EE(\%)=\frac{m_{0}-m_{1}}{m_{0}}\times 100$$

where m_0_ (g) was the total weight of metolachlor in the reaction system and m_1_ (g) was the total weight of non-encapsulated metolachlor in the reaction system. And m_1_ was calculated as Equation (3).

(3) $$m_{1}=\frac{m_{3}\times m_{4}}{m_{2}}$$

where m_2_ (g) was the weight of microcapsules suspension added in column, m_3_ (g) was the total weight of the reaction system in a three-necked flask, m_4_ (g) was the weight of metolachlor detected from m_2_.

### 3.5. Thermogravimetric analysis

The thermal analyses of microcapsules were carried out on a DSC analyzer (DSC-204,NETZSCH). All experiments were carried out with a sample whose weight was about 5 mg with a heating rate of 15°C/min and nitrogen as the purging and protective gas. Each sample was analyzed at least twice and the average value was recorded. The thermal stability characterization was performed on a TGA analyzer at a heating rate of 15°C/min among the range of 30–750°C in a flow of 60 mL/min nitrogen [19].

## 4. Results and discussion

### 4.1. FT-IR analysis of microcapsules

**Figure 4.**
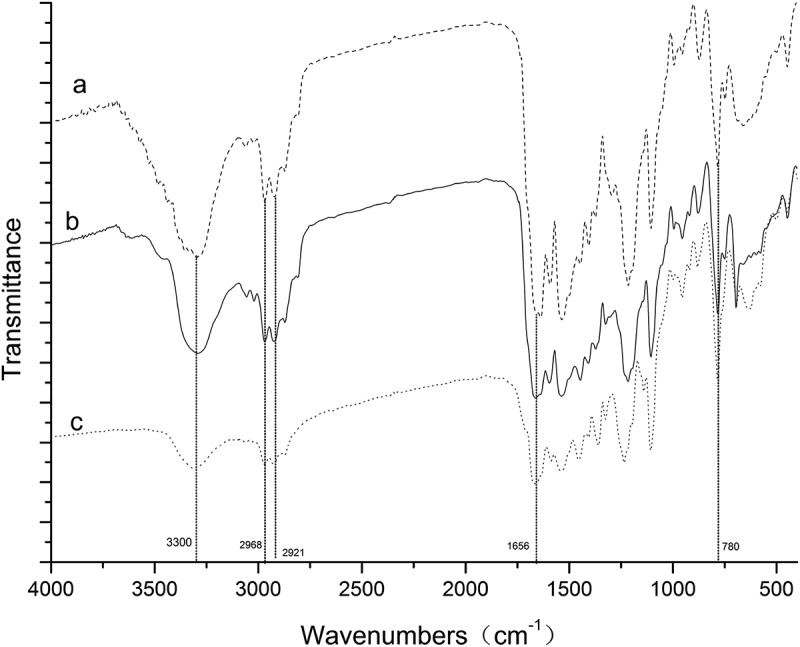
FTIR spectra of polyurea microcapsules from (a) DETA, (b) Urea, and (c) EDA.

Figure 4 shows the FTIR spectra of polyurea microcapsules from different amines of DETA, Urea, and EDA. All polyurea microcapsules in these experiments have strong bands known for hydrogen bonded N-H stretching vibration at 3300 cm^−1^ were observed. The characteristic peaks appearing at 2968 cm^−1^ and 2921 cm^−1^ are due to the C-H stretching vibrations of aliphatic diamine. The absorption peak at 1656 cm^−1^ and 780 cm^−1^ were contributed by C = O stretching vibration and C-Cl stretching vibration, respectively. The IR spectrum also showed that the reaction between the diisocyanate and the diamine was completed by the disappearance of the NCO absorption band at 2270 cm^−1^ and the appearance of the N-H and C = O absorption bands. All these characteristic absorption peaks of metolachlor could also be found in the spectrum of the polyurea microcapsules. From these characteristic peaks, it could be concluded that the core and wall materials of the prepared microcapsules were metolachlor and polyurea, respectively.

### 4.2. Particle size and particle size distribution

**Figure 5.**
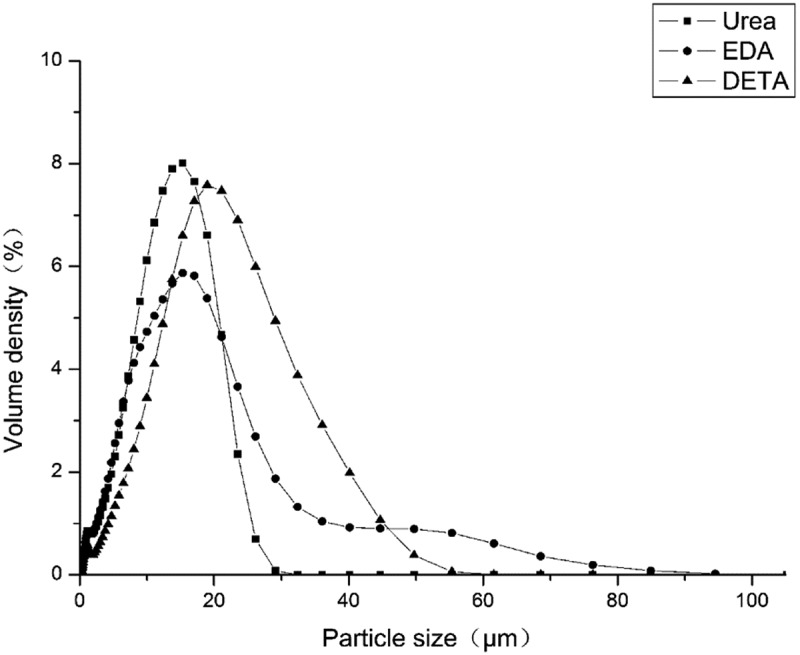
Particle size distribution of polyurea microcapsules with metolachlor.

As discussed earlier, the microcapsules are produced via emulsification followed by interfacial polymerization of the wall-formers. Therefore, the particle size and size distribution of the microcapsules are a fundamental factors affecting the surface area and core content [20,21]. Microcapsule size is one of the important factors governing the release rate of microcapsules containing metolachlor. In this work, size distribution and particle size were measured by a laser particle size analyzer. Figure 5 and Table 2 shows the particle size distribution of the prepared polyurea microcapsules containing metolachlor. The microcapsules mean size (based on volume distribution) is 10–20 μm. Figure 5 shows the particle size distribution curves for the polyurea microcapsules prepared with different aqueous monomers where stirring rate was set at 2000 rpm for about 5 min during the emulsion preparation. A broad size distribution of microcapsules was found, covering approximately 1–100 μm. The mean diameters were 11.52, 11.88 and 17.16 μm for microcapsules obtained with urea, DETA and EDA. The size distribution corresponds to the results of SEM photographs shown in Figure 8. As it were, a mean particle size is the smallest and the size distribution is the narrowest for the sample from Urea. In the cases of the samples from EDA and DETA, aggregation among the globules resulted in much broader size distribution. This is related to the formation of much greater agglomerates by broken globules. This is related to differences in wall thickness due to reactivity of urea or polyamine used [22,23].

**Table 2.** The D_50_ and spans of three microcapsule samples.

Samples	Aqueous monomers	D_50_(μm)	Span
a	Urea	11.52 ± 0.28	1.55 ± 0.12
b	DETA	11.88 ± 0.34	1.67 ± 0.16
c	EDA	17.16 ± 0.36	2.16 ± 0.22

### 4.3. Encapsulation efficiency analysis

**Figure 6.**
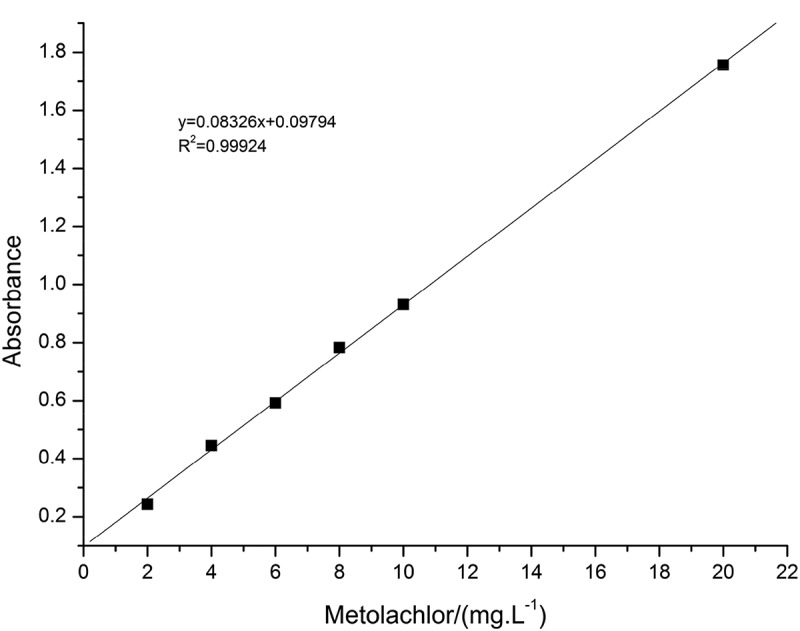
The relationship between the concentration of metolachlor and the absorbance intensity at 266 nm.

The standard curve is shown in Figure 6. As shown in Figure 6, we could see that the correlation coefficient between the concentration of metolachlor and the absorbance intensity is 0.99924. The function relation is y = 0.08326x+0.09794.

**Figure 7.**
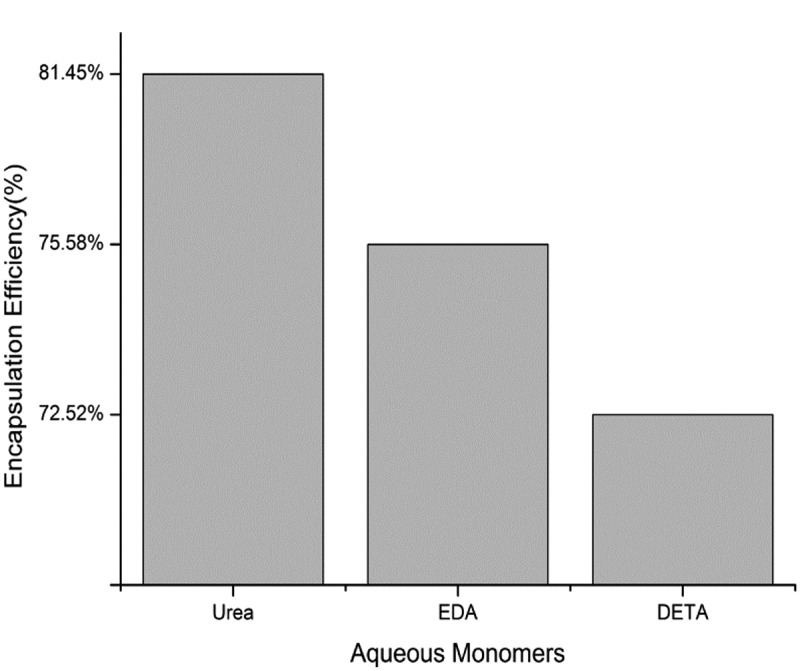
Encapsulation efficiency of microcapsules with different aqueous monomers.

Figure 7 presents the encapsulation efficiency of microcapsules with different aqueous monomers. When urea was used as a water-soluble monomer, the polyurea microcapsules produced had the highest level of encapsulation efficiency (81.45%). The microcapsules were prepared using the other two polyamines have lower encapsulation efficiency. The encapsulation efficiency of polyurea microcapsules were prepared using EDA and DETA was 75.58% and 72.52%, respectively. As shown in Figure 7. The polyurea microcapsules were prepared by replacing polyamine (EDA or DETA) with urea had better encapsulation efficiency, which is 5.87% and 8.93% higher than that of the two kinds of microcapsules and the encapsulation efficiency is remarkably improved.

### 4.4. Morphology of microcapsules

**Figure 8.**
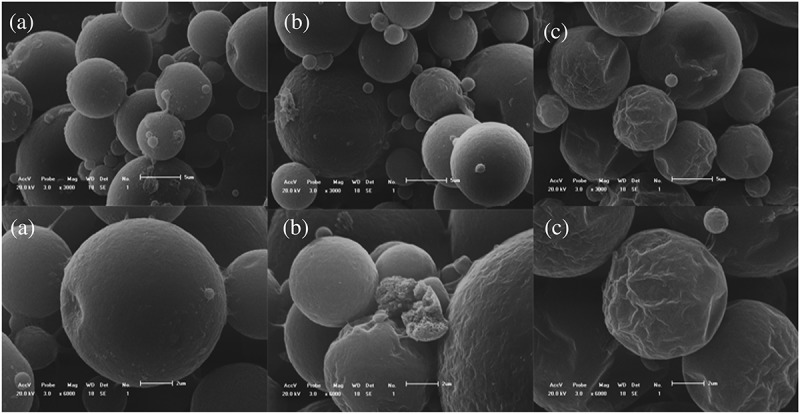
SEM micrographs of (a) Urea, (b) EDA, and (c) DETA.

Observing the morphology of microcapsules has great significance to the research of micro-encapsulation [24]. The scanning electron microscope (SEM) images of polyurea microcapsules containing metolachlor were shown in Figure 8. As shown in the pictures. It was visualized that the prepared polyurea microcapsules were globular-shaped. The rupture of the shell can be clearly seen in Figure 8(b). There appeared to be holes rather big in scale on the surface of microcapsules, causing incompleteness of the microcapsules, and the fragments of the microcapsule wall dispersed around the microcapsule itself. The reason for this is that harsh process condition of sharp mixer blade and weak membrane strength [25,26]. Figure 8(c) shows the prepared polyurea microcapsules with diethylenetriamine. The microcapsules were spherical without breaking microcapsules, but the surface of the microcapsules was wrinkled and had obvious depressions. Shrinkage appeared in SEM of microcapsules would have been because of the reduction in internal area which might be due to release of solvent and core material that made availability of internal free volume for shrinkage of wall material. As per the literature, wrinkling may be resulted due to the interaction of inhomogeneous reaction kinetics, fluid-induced shear forces, and shell-determined elastic forces [27]. As shown in Figure 8(a), the SEM images revealed that the urea microcapsules were almost spherical, compact outer surface, and no ruptured phenomenon. These microcapsules had a highly centralized particle size distribution, a high quality with minimal impurity and a high production rate.

### 4.5. Thermogravimetric analysis

When self-healing composites are thermally cured, the embedded microcapsules have to experience heat treatment. The thermal stability plays an important role in their application [27–29].

**Figure 9.**
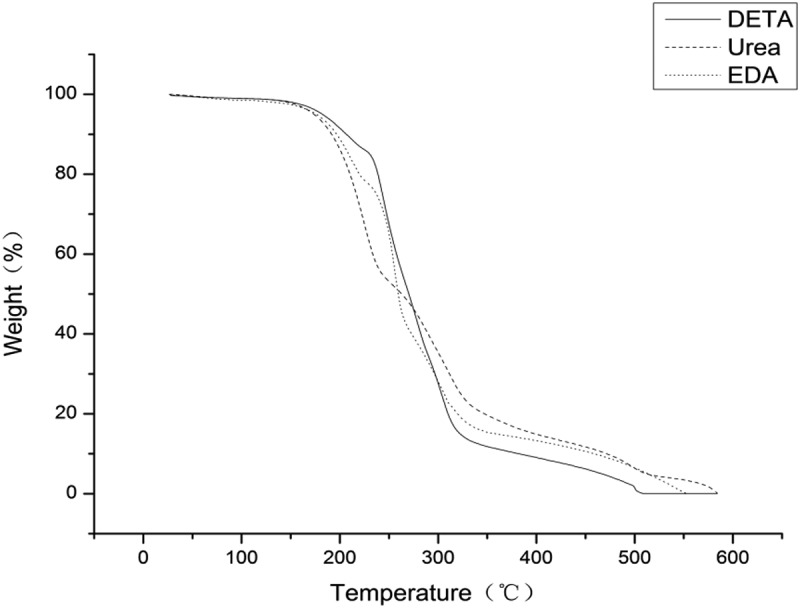
TGA thermograms of polyurea microcapsules from (a) Urea, (b) DETA, and (c) EDA.

Figure 9 presents TGA diagrams of polyurea microcapsules from different water soluble monomers. Weight loss of each sample also verifies the formation of polyurea microcapsules with high thermal stability [30,31]. As shown in the figure, it can be seen that there are three main phases of weight loss for the polyurea microcapsules within the temperature range of interests. The first phase with 5% weight loss from 25 to 100°C should be due to evaporation of the absorbed water. The second stage of weight loss of about 73–82% in the range of 100–330°C is attributed to the volatilization and decomposition of the core material metolachlor. The third phase with about 13–22% weight loss in the range of 270–600°C originates from decomposition of polyurea wall materials [12,32].

As shown in Figure 9, the mass loss process of metolachlor begins at 160°C and ends at 330°C, which are corresponding to the volatilization of metolachlor. In addition, the initial temperature loss of metolachlor is much lower than that of polyurea wall materials, which prove that the thermal stability of polyurea wall materials is much better than that of pure metolachlor. In the third stage, it could be seen that the polyurea microcapsules prepared by using urea instead of the polyamine were completely decomposed at 580°C, and the microcapsules prepared by using DETA and EDA were completely decomposed at 505°C and 550°C, respectively. This also proved that polyurea microcapsules prepared using urea have higher thermal stability. This might be induced from the differences in the structure of the polymer wall prepared using different water soluble monomers [33].

These results confirmed that metolachlor was encapsulated by the polyurea shell. When urea was used as a water-soluble monomer to prepare polyurea microcapsules, the microcapsules had higher thermal stability and could be better preserved in polyurea microcapsules. It could also be helpful to reduce the degradation of core material for its longer and efficient utilization [34,35].

## 5. Conclusions

Metolachlor-containing polyurea microcapsules were prepared by the interfacial polycondensation of TDI and different water soluble monomers, Urea, EDA, DETA. When we observed the spectra of microcapsules by FTIR, we could clearly observe the formation of polyurea structure. The results indicated that it is feasible to prepare polyurea microcapsules by using urea instead of polyamines. The Particle size distributions and encapsulation efficiency of polyurea microcapsules were studied by particle size analyzer and ultraviolet spectroscopy. We found that urea microcapsules had a smaller particle size (11.52 μm) and an optimum encapsulation ratio (81.45%) which might be related to the thickness of the formed wall formed by the reaction of urea with TDI. Urea microcapsules had the best thermal stability and would completely decompose at 580°C. They could be kept at room temperature for a long time and could bear the moderate temperature or high temperature applied for curing composites. This indicated that the use of urea synthesis of polyurea microcapsules could better preserve isopropyl methachlor. In addition, it greatly reduced the use of organic solvents and facilitates environmental protection in the process of the microcapsule preparation. It is very important significance to reduce products cost and pollution by employing the technology. It also could provide new ideas and methods for the synthesis of agrochemicals and environmental protection. It is hoped that this polyurea material and microcapsules are used in the field of material chemistry or other fields.
